# The Impact of Package Type and Temperature on the Changes in Quality and Fatty Acids Profile of Table Eggs during Their Storage

**DOI:** 10.3390/foods10092047

**Published:** 2021-08-31

**Authors:** Kamil Drabik, Tomasz Próchniak, Damian Spustek, Karolina Wengerska, Justyna Batkowska

**Affiliations:** Institute of Biological Basis of Animal Production, University of Life Sciences in Lublin, 13 Akademicka St., 20-950 Lublin, Poland; kamil.drabik@up.lublin.pl (K.D.); tomasz.prochniak@up.lublin.pl (T.P.); damianspustek@gmail.com (D.S.); karolina.wengerska@gmail.com (K.W.)

**Keywords:** chicken eggs, cardboard egg box, plastic egg box, fridge temperature, room temperature, egg storage, fatty acid profile

## Abstract

The aim of the study was to evaluate the possibility of reducing changes in the quality of consumer hen eggs by storing them in various package type and under various temperature conditions (room and refrigeration). The material consisted of 960 chicken eggs packed in cardboard or plastic boxes, 10 pcs in each. Half of the packages were stored at room temperature (21 °C), the rest in the refrigerator (5 °C). The eggs were stored for 28 days qualitatively evaluated at 14-day intervals. The characteristics of whole egg (weight, specific weight, proportion of morphological elements, air cell depth) as well as of shell (weight, color, crushing strength, thickness, density, water conductivity), albumen (height, Haugh units, weight, pH) and yolk (weight, color, pH) were analyzed. The fatty acids profile of yolks was also evaluated as a freshness indicator. Packaging types available on the market, apart from its marketing and eggs protection function, can also influence the quality and stability of the product during storage. The use of plastic boxes can help to maintain higher eggs quality during the storage period, even after a significant extension of the storage time. Eggs stored in plastic boxes at room temperature had very similar results to those stored under refrigeration using conventional cardboard boxes. This effect is probably related to the lower permeability of plastic boxes in comparison to cardboard ones, but detailed research work in this direction is necessary to verify this relation.

## 1. Introduction

Eggs, due to their balanced chemical composition and low price are one of the most important animal products in the human diet. Therefore, solutions are being sought to obtain eggs of the best quality or with improved nutritional value. The vast majority of works in this field focus on the modification of laying hen nutrition to increase the quality and improve the chemical composition of the obtained eggs [1,2].

At the same time, like all products available on the market, table eggs must meet the consumers’ requirements. Analyzing data from available literature Rondoni et al. [3] pointed out that purchasing behavior is closely related to the place of research, but there are also common elements regarding egg appearance, freshness or packaging.

In addition to factors related to the bird housing system or egg appearance, one of the most important criteria for the selection of table eggs is their freshness [4,5]. Regardless of the origin of the eggs, with the time of storage, there is a deterioration of their quality and technological usefulness. In the EU, the marketing of table eggs is regulated by Commission and Council Regulation (EC) 589/2008 [6], which introduces a 28-day shelf life for Class A table eggs.

Moreover, this regulation limits the possibility of refrigerated storage of table eggs at the commercial stage, indicating such a possibility only for final consumers. Despite the positive effect of refrigeration methods on inhibiting negative changes in the quality of table eggs confirmed in studies [7,8], it is necessary to search for alternatives in this regard. So far, two main methods have been developed: coating egg shells with substances that limit water evaporation through shell pores [9,10,11,12] or using atmospheric modification [13]. These methods, although effective, are not currently of applicable importance.

Another element that may inhibit changes in the quality of consumer eggs is the type of packaging in which they were purchased. In the vast majority of cases consumer eggs are packed in small boxes (6 or 10 pieces each). Both cardboard and plastic packages are available on the market. It is, therefore, reasonable to find out whether the type of packaging (cardboard vs. plastic) is important in terms of egg storage.

The study aimed to evaluate the possibility of reducing changes in the quality of consumer hen eggs by storing them in various package types (cardboard or plastic egg box) and under various temperature conditions (room and refrigeration).

## 2. Materials and Methods

### 2.1. Material Preparation

The material for the study consisted of 960 L-class table eggs purchased from the laying hens stock kept in a cage system. Eggs were collected on the same day, numbered individually and then randomly divided into 4 treatments according to the scheme (Table 1). The quality of 60 eggs, treated as a control group was also evaluated on the same day (day 0), the rest of the eggs were stored in 10-piece egg boxes, made of plastic and/or cardboard, divided into two storage temperatures, room (21 °C) and refrigerated (5 °C).

### 2.2. Egg Quality Analyses

Egg quality was analyzed at 14-day intervals. An EQM (Egg Quality Measurement, TSS^®^, York, UK), Instron Mini 55 device (Instron^®^, Norwood, MA, USA) and pH-meter with combined glass electrode (Elmetron^®^, Zabrze, Poland) were used. The following experimental material characteristics were evaluated:

- whole egg—depth of the air cell (ACD), mass (EW), proportions of morphological elements (in relation to egg weight, EYP—yolk proportion in egg weight, EAP—albumen proportion in egg weight, ESP—shell proportion in egg weight).
- shell–colour (SC), weight (SW), thickness (ST), strength (SS), eggshell proportion egg weight.
- albumen–weight (AW), height (AH), pH (ApH).
- yolk–weight (YW), colour (YC, using 16-points DSM YolkFan^TM^, DSM Nutritional Products, Basel, Switzerland), index (YI, as ratio of its height and diameter), pH (YpH).

Additional quality parameters, such as weight loss (WL) and specific mass of eggs (ESG, according to Archimedes principle), shell density (SD) [14] and its water vapor conductance (ESC) (Ar et al., 1974) and Haugh’s units (HU) [15] were calculated based on the obtained data.

### 2.3. Yolk Lipid Profile Analyses

On the day of the experiment, and after 28 days, 20 yolks/treatment were collected for further analyses. The samples were freeze-dried (Labconco Corporation, Kansas City, MO, USA), and then fatty acid profiles and cholesterol content were analyzed.

The fatty acid profile of egg yolks was analyzed using gas chromatography according to PN-EN ISO 5508: 1996 and PN-EN ISO 5509: 2001. The Varian 450-GC gas chromatograph (Agilent Technologies, Santa Clara, CA, USA) with the flame ionization detector (FID) equipped with capillary column Select™ Biodiesel for FAME (30 m 0.32 mm 0.25 μm) and autosampler CP-8400 were used for the analyses. Based on the proportions of particular fatty acids and their groups (Galaxie™ Chromatography Data System software), the following indexes were calculated: PI-peroxidizability index [16], AI –atherogenicity index and TI-thrombogenic index [17], DFA-desirable fatty acids [18], HFSA-hypercholesterolaemic saturated fatty acids [19] and h/H—hypocholesterolaemic/hypercholesterolaemic ratio [20].

### 2.4. Statistical Analyses

The data obtained were statistically analyzed using the SPSS 24.0 statistical package [21]. The Shapiro-Wilk test was used to assess the normal distribution of the traits. A two-factorial model of analysis of variance was then used, taking into account the type of packaging (P-cardboard or plastic), storage temperature (T-room or fridge) and the interaction between them. Group comparisons were made using Tukey’s multiple comparisons test.

## 3. Results

### 3.1. Egg Quality

With the storage time of eggs, all their quality characteristics change. Table 2 shows the parameters of eggs external traits. Although all the eggs were classified as “L”, they were slightly different in terms of weight, which explains the differences observed in the initial phase of the experiment. After 42 days of storage, it was found that eggs stored in plastic boxes at refrigeration temperature had significantly the highest weight. Observations on egg specific gravity (ESG) showed no significant differences after 28 days of storage regardless of the packaging used. The extended storage period showed that storage in plastic boxes allowed to inhibit changes in this range.

Similar observations also relate to egg weight loss during the storage. After 28 days, it was found that the lowest losses, regardless of storage temperature, were characteristic of eggs stored in plastic boxes. Prolongation of storage time indicated the continuation of this trend, with the highest weight loss registered in eggs stored at room temperature in cardboard boxes. As water loss is closely related to shell water conductivity, the data obtained for both 28 and 42 days of storage of table eggs indicate the same relationship as for WL.

Air cell depth (ACD) differed significantly between the experimental groups after both 28 and 42 days of storage. Significantly lower values in this regard were recorded for eggs stored in plastic boxes compared to those made of cardboard. At the same time, it should be noted that after 42 days of storage only in the case of eggs from the RCB group the air cell depth exceeded 6 mm, i.e., the limit value for consumer eggs of class A, was recorded.

Changes in egg quality also affected its content. It was found that the highest weight of albumen after 42 days of storage characterized eggs from the FPB group (Table 3). A similar trend was also observed for the albumen proportion in the whole egg weight.

It was also observed that albumen height and Haugh’s unit number decreased during eggs’ storage, regardless of the packaging type of temperature applied. At the same time, it should be noted that for both AH and HU the highest quality was found of eggs stored at refrigeration temperature in plastic packaging s, while the lowest values for both traits were recorded for the RCB group. Importantly, eggs stored at room temperature in plastic packs had better quality compared to those stored under refrigeration in cardboard packs. 

In terms of changes in albumen pH, it was found that eggs from the RPB and FPB groups had a lower pH only after 14 days of the experiment compared to those stored in cardboard packaging, and this trend was maintained until the end of the storage time. Although there were no significant differences in yolk weight during egg storage (Table 4), the proportion of this egg morphological element, after 42 days of the experiment was considerably the lowest in the FPB group, while the other groups did not differ statistically. 

The yolk shape index decreased during storage regardless of temperature or type of packaging. After 28 days of storage, it was found that eggs from groups stored in plastic boxes were characterized by significantly higher shape index values compared to those stored in cardboard ones.

The yolk pH was also significantly affected by time. After 28 days of the experiment, eggs from the FPB group had the lowest values, while the other study groups did not differ significantly.

In terms of egg quality, it was found that almost all whole egg traits (Table 2) were significantly influenced by temperature, type of packing as well as the interaction of both factors. Slightly different observations were made for egg content quality traits (Table 3 and Table 4). Most of them remained significantly influenced by the storage temperature. Definitely fewer traits showed a dependence on the type of packaging and the interaction of both experimental factors.

### 3.2. Fatty Acids Profile

From the yolk samples taken at the time of analysis, the fatty acid composition was determined (Table 5) and their indices were calculated (Table 6). None of the saturated fatty acids (SFA) showed variation with temperature or type of packaging. Similar observations were also made for monounsaturated fatty acids.

Among PUFAs, 3 polyunsaturated fatty acids were found to change with storage time. Among them, arachidonic acid (C20:4 n6) and docosahexaenoic acid (C22:6 n3) remained at significantly highest levels in egg yolks stored under refrigeration in plastic boxes compared to the other experimental groups.

Concerning fatty acid indices, significant differences were observed only in the case of the n3 group (Table 6). The highest content of n3 fatty acids was found in yolks of eggs stored in refrigerator (FCB and FPB). The values obtained for these groups did not differ significantly from those obtained for fresh eggs (0 days).

## 4. Discussion

Natural changes in the raw material of eggs are the result of biophysical and chemical changes taking place in the egg content from the moment when the eggs are laid. However, although time is one of the basic elements influencing changes in the quality of table eggs, factors related to the egg itself or environmental conditions are also important. Egg weight loss during storage occurs regardless of the environmental conditions or protective treatments applied [9,22,23,24]. At the same time, it should be noted that the intensity of these changes can be reduced by decreasing the intensity of gas exchange between the external environment and the egg contents. The most common practice is to moderate the storage temperature, which reduces the loss of egg mass. Several studies indicate the effectiveness of this method [8,25], which is also confirmed in our research. Interestingly, the type of packaging also significantly contributed to the inhibition of egg weight loss or air chamber deepening, allowing similar results for cardboard boxes for refrigeration temperature and plastic ones at room temperature. This was probably due to the difference in the structure of the material, thus greater access of air to eggs stored in cardboard boxes. These observations seem all the more relevant given that the legislation adopted in the EU reserves the refrigerated storage of eggs to the final consumer only. The use of suitable packaging, which is already available on the market, could therefore be an alternative to reduce the storage temperature of the egg raw material.

The reduction of the weight loss of eggs during storage also contributed to the inhibition of other changes in their quality. One of the basic characteristics analyzed in this respect is the air cell depth. The changes in this parameter observed in our study are similar to those noticed in previous studies [8]. At the same time, it should be emphasized that the limit value of 6 mm [6] was exceeded only in the case of eggs stored for 42 days at room temperature in cardboard boxes, while in the remaining cases, even the extended period of storage did not influence its deepening to a higher degree than assumed in the legislation.

Quality changes also affect particular elements of the egg content. With time, there is a loss of albumen mass both through evaporation and the diffusion that occurs from the albumen to the yolk [26]. These changes entail further changes, such as decreases in albumen height or related to Haugh units. Early studies [27] indicated that it is the loss of albumen mass that is one of the most important factors in changing its structure. At present, an equally important role is attributed to albumen alkalisation through the release of carbon dioxide as a result of carbonic acid dissociation [28], which in turn leads to the weakening of bonds within the ovomuccin-lysosome complex, which is one of the factors responsible for maintaining the correct structure of dense albumen. Our studies, and those presented by other authors, agree on the role of time in the occurrence of these changes, as well as proving that lowering the storage temperature can effectively reduce their intensity [7,29].

Quality changes during egg storage also affect the yolk. Both our own studies and reports by other authors indicate an increase in yolk mass by diffusion of water from the albumen [26], but also an increase in the proportion of egg mass or a decrease in the shape index value [30]. Since these changes adversely affect the quality of the raw materials obtained, it is necessary to inhibit these processes. One of the most popular methods in this respect is lowering the storage temperature, which at the same time is one of the most effective, which is confirmed by the study of Keener et al. [25], as well as the results obtained by us. It should also be noted that the use of plastic boxes proved to be even more effective than lowering the temperature for eggs stored in classical cardboard boxes.

Apart from changes in quality characteristics and those affecting the technological value of eggs, their chemical composition may also change during storage. Since egg yolk is a valuable source of polyunsaturated fatty acids, lipid peroxidation during storage is a particularly important issue in this respect. The high UFA content in yolk makes it all the more vulnerable to oxidation [31]. With this in mind, it is necessary to ensure that the oxidative stability of the harvested eggs is as high as possible. Many works focus in this respect on the use of feed additives of antioxidant nature such as cinnamaldehyde [32] or plant extracts of thyme or oregano [33]. In our study, no similar additives were used and the study material was unified, so the variability observed was solely due to the storage methods used.

The available literature does not provide information on the effect of storage on changes in the fatty acid profile of egg yolks. Admittedly, some works indicate that dimaldehyde content rises with storage time and temperature [33], while according to other authors similar differences are almost imperceptible [34]. Although in our study the level of MDA was not analyzed, the observed differences in the concentration levels of arachidonic acid and ALA may indicate the occurrence of lipid oxidation [33]. Regardless of the background of this variation, it was found that apart from lowering the temperature, also the use of plastic boxes reduces the intensity of these changes.

The fatty acid profile provides valuable nutritional and health-promoting information, however, in terms of consumer safety of the eggs, fatty acid indices are considerably better. The most commonly assessed are the SFA/PUFA ratio and the much more accurate AI and TI, which determine indices of atherogenicity and thrombogenicity respectively [17].However, these indices are mostly mentioned in the context of changes occurring in eggs due to supplementation of birds with various lipid-like substances, e.g., natural vegetable oils [35,36].The number of studies analysing such changes over time is very limited. The results obtained in our study for fatty acid indices for term 0 are partly in line with those described by other authors, such as Attia et al. [37], who show that eggs originating from different purchase locations, were characterized by significant variability for this trait. These observations may explain the differences between the results of our study and those presented for control groups (i.e., birds fed a standard feed mixture) by other authors. For example, Omari et al. [38] found similar relationships between particular indices, but the results presented by them differ significantly from those obtained in our study. As the subject of the study was the type of packaging and storage temperature of the raw material, it should be noted that the only differences were observed in the case of PUFAs and especially the group of n3 acids, i.e., those susceptible to oxidation [39]. In the case of other indices, no significant differences were found, which may suggest that in the case of variation in the profile of fatty acids and their indices, storage is a factor of relatively minor importance in the case of shelled eggs.

## 5. Conclusions

Packaging types available on the market, apart from its marketing and eggs protection function, can also influence the quality and stability of the product during storage. Research has shown that the use of plastic boxes can help to maintain higher eggs quality during the storage period, even after a significant extension of the storage time.

The study showed that whole egg characteristics (weight, specific gravity, air cell depth) changed significantly less in groups stored at refrigeration temperature, but it was also found that a similar effect could be obtained at room temperature using plastic egg boxes. Despite the prolonged storage time and varied storage conditions, it was found that storage had little effect on the fatty acids profile and their indices, and the only change over time was observed for PUFAs (mainly n3), which content changed in the least in the group of eggs stored in plastic boxes and at reduced temperature.

Importantly, eggs stored in plastic boxes at room temperature had very similar results to those stored under refrigeration using conventional cardboard boxes. This gives real hope for their use on a wider scale as an alternative to refrigerated storage, which in the EU is reserved exclusively for the final consumer. This effect is probably related to the lower permeability of plastic boxes in comparison to cardboard ones, but detailed research work in this direction is necessary to verify this relationship.

## Figures and Tables

**Table 1:** Schema of the experiment.

Package Type	Cardboard Box (CB)		Plastic Box (PB)	
Temperature	Room (R, 21 °C)	Fridge (F, 5 °C)	Room (R, 21 °C)	Fridge (F, 5 °C)
Time (Days)	RCB	FCB	RPB	FPB
0	60			
14	60	60	60	60
28	60	60	60	60
42	60	60	60	60
Total	240	240	240	240

RCB—cardboard box, room temperature (21 °C); FCB—cardboard box (5 °C), refrigeration temperature (5 °C), RPB—plastic box, room temperature (21 °C), FPB—plastic box, refrigeration temperature (5 °C).

**Table 2:** Changes in the characteristics of the whole egg depending on packaging and storage temperature.

Trait	Time (Days)	Treatment				Total	SEM	Factor (*p-*Value)		
		RCB	FCB	RPB	FPB			B	T	B × T
EW (g)	0	63.29 ^a^	63.36 ^a^	63.30 ^a^	64.83 ^b^	63.66	0.159			
	14	62.36 ^a^	63.10 ^ab^	63.11 ^ab^	64.49 ^b^	63.27	0.268	*0.042*	*0.041*	*0.187*
	28	61.56 ^a^	61.85 ^a^	62.34 ^ab^	64.31 ^b^	62.51	0.263	*0.020*	*0.001*	*0.080*
	42	59.95 ^ab^	59.16 ^a^	60.71 ^b^	64.08 ^c^	60.92	0.297	*0.004*	*0.000*	*0.000*
ESG (g/cm^3^)	0	1.079					0.002			
	14	1.059 ^a^	1.072 ^b^	1.081 ^c^	1.081 ^c^	1.073	0.001	*0.007*	*0.000*	*0.000*
	28	1.078	1.054	1.072	1.076	1.070	0.011	*0.001*	*0.713*	*0.536*
	42	1.019 ^a^	1.043 ^b^	1.067 ^c^	1.070 ^c^	1.049	0.003	*0.001*	*0.000*	*0.020*
WL (%)	14	4.37 ^c^	3.27 ^b^	2.61 ^a^	2.22 ^a^	3.15	0.112	*0.000*	*0.000*	*0.006*
	28	6.56 ^c^	4.33 ^b^	3.00 ^a^	2.87 ^a^	4.15	0.215	*0.000*	*0.000*	*0.000*
	42	8.47 ^c^	5.39 ^b^	3.25 ^a^	3.41 ^a^	5.22	0.279	*0.000*	*0.000*	*0.000*
ESC (mg/day/torr)	14	2.28 ^c^	1.73 ^b^	1.37 ^a^	1.17 ^a^	1.64	0.057	*0.000*	*0.000*	*0.008*
	28	1.73 ^c^	1.08 ^b^	0.77 ^a^	0.75 ^a^	1.07	0.056	*0.000*	*0.000*	*0.000*
	42	1.48 ^c^	0.91 ^b^	0.54 ^a^	0.60 ^a^	0.90	0.049	*0.000*	*0.000*	*0.000*
ACD (mm)	14	3.83 ^c^	2.80 ^b^	2.10 ^a^	2.23 ^ab^	2.74	0.109	*0.005*	*0.000*	*0.000*
	28	5.20 ^c^	3.98 ^b^	2.80 ^a^	2.55 ^a^	3.63	0.159	*0.001*	*0.000*	*0.028*
	42	6.33 ^c^	4.52 ^b^	4.20 ^a^	3.40 ^a^	4.63	0.206	*0.001*	*0.000*	*0.172*

^a,b,c^—means within row differ significantly at *p* ≤ 0.05; SEM—standard error of mean; RCB—(21 °C); FCB—cardboard box (5 °C), refrigeration temperature (5 °C), RPB—plastic box, room temperature (21 °C), FPB—plastic box, refrigeration temperature (5 °C); B—egg box type, T—temperature; EW—egg weight, ESG—egg shell gravity, WL—weight loss, ESC—water vapor conductance, ACD—air cell depth.

**Table 3:** Changes in albumen characteristics concerning packaging and storage temperature.

Trait	Time (Days)	Treatment				Total	SEM	Factor (*p*-Value)		
		RCB	FCB	RPB	FPB			B	T	B × T
AW (g)	0	41.12					0.628			
	14	36.55	38.88	38.49	39.07	37.72	0.630	*0.048*	*0.994*	*0.886*
	28	36.43 ^a^	38.28 ^ab^	38.03 ^ab^	39.14 ^b^	37.97	0.352	*0.033*	*0.076*	*0.592*
	42	34.89 ^a^	34.46 ^a^	35.15 ^a^	39.38 ^b^	36.07	0.391	*0.002*	*0.000*	*0.000*
EAP (%)	0	63.77					0.548			
	14	58.61	60.63	60.06	60.56	59.10	0.929	*0.110*	*0.575*	*0.601*
	28	59.12	61.93	60.94	60.86	60.71	0.476	*0.152*	*0.693*	*0.129*
	42	58.27 ^a^	56.95 ^a^	57.93 ^a^	61.46 ^b^	58.75	0.326	*0.013*	*0.000*	*0.000*
AH (mm)	0	8.25					0.385			
	14	5.64 ^a^	4.74 ^a^	7.20 ^b^	7.34 ^b^	6.23	0.228	*0.335*	*0.000*	*0.188*
	28	3.08 ^a^	3.68 ^a^	6.74 ^b^	6.60 ^b^	5.02	0.213	*0.275*	*0.000*	*0.081*
	42	2.51 ^a^	2.96 ^a^	4.41 ^b^	6.47 ^c^	4.01	0.206	*0.000*	*0.000*	*0.000*
HU	0	88.67					2.124			
	14	68.17 ^a^	63.32 ^a^	83.12 ^b^	83.46 ^b^	74.51	1.665	*0.406*	*0.000*	*0.341*
	28	45.08 ^a^	52.68 ^b^	80.54 ^c^	78.81 ^c^	64.28	2.021	*0.152*	*0.000*	*0.024*
	42	35.08 ^a^	42.34 ^a^	61.06 ^b^	78.32 ^c^	53.13	2.417	*0.091*	*0.000*	*0.000*
ApH	0	8.51					0.075			
	14	9.19 ^b^	9.13 ^b^	8.95 ^a^	8.88 ^a^	9.04	0.023	*0.026*	*0.000*	*0.765*
	28	9.28 ^b^	9.20 ^b^	8.86 ^a^	8.95 ^a^	9.09	0.032	*0.899*	*0.000*	*0.054*
	42	9.12 ^b^	9.05 ^b^	8.82 ^a^	8.74 ^a^	8.95	0.030	*0.200*	*0.000*	*0.136*

^a, b, c^—means within row differ significantly at *p* ≤ 0.05; SEM—standard error of mean; RCB—cardboard box, room temperature (21 °C); FCB—cardboard box (5 °C), refrigeration temperature (5 °C), RPB- plastic box, room temperature (21 °C), FPB—plastic box, refrigeration temperature (5 °C); B—egg box type, T—temperature; AW—albumen weight, EAP—albumen proportion in egg weight, AH—albumen height, HU—Haugh’s units; ApH—albumen pH.

**Table 4:** Changes in the characteristics of yolk depending on packaging and storage temperature.

Trait	Time (Days)	Treatment				Total	SEM	Factor (*p-*Value)		
		RCB	FCB	RPB	FPB			B	T	B × T
YW (g)	0	15.50					0.236			
	14	17.87	17.02	16.96	17.18	17.80	0.551	*0.206*	*0.516*	*0.609*
	28	16.89	15.62	16.14	16.90	16.39	0.278	*0.646*	*0.634*	*0.072*
	42	17.17	17.52	17.42	16.58	17.15	0.160	*0.173*	*0.615*	*0.059*
EYP (%)	0	24.10					0.437			
	14	28.64	26.57	26.52	26.65	27.97	0.242	*0.134*	*0.686*	*0.719*
	28	27.48	25.22	25.95	26.28	26.23	0.457	*0.295*	*0.800*	*0.161*
	42	28.67 ^b^	29.70 ^b^	28.69 ^b^	25.86 ^a^	28.16	0.295	*0.019*	*0.001*	*0.000*
YC (pkt.)	0	12.10					0.376			
	14	11.55 ^ab^	10.75 ^a^	11.50 ^ab^	11.70 ^b^	11.38	0.130	*0.236*	*0.077*	*0.050*
	28	11.45 ^ab^	11.15 ^a^	11.95 ^ab^	12.00 ^b^	11.64	0.121	*0.593*	*0.005*	*0.455*
	42	10.05 ^ab^	9.83 ^a^	11.31 ^b^	11.15 ^ab^	10.53	0.197	*0.283*	*0.000*	*0.600*
YI	0	0.414					0.009			
	14	0.362 ^a^	0.365 ^a^	0.402 ^b^	0.400 ^b^	0.383	0.006	*0.949*	*0.002*	*0.814*
	28	0.275 ^a^	0.279 ^a^	0.390 ^b^	0.385 ^b^	0.331	0.009	*0.931*	*0.000*	*0.667*
	42	0.258 ^a^	0.267 ^a^	0.359 ^b^	0.405 ^c^	0.325	0.010	*0.340*	*0.000*	*0.000*
YpH	0	6.12					0.012			
	14	6.21	6.25	6.17	6.18	6.20	0.020	*0.585*	*0.221*	*0.673*
	28	6.46 ^b^	6.32 ^b^	6.35 ^b^	6.06 ^a^	6.30	0.034	*0.001*	*0.003*	*0.179*
	42	6.50 ^b^	6.54 ^b^	6.35 ^ab^	6.25 ^a^	6.42	0.030	*0.451*	*0.000*	*0.064*

^a,b,c^–means within row differ significantly at *p* ≤ 0.05; SEM—standard error of mean; RCB—cardboard box, room temperature (21 °C); FCB—cardboard box (5 °C), refrigeration temperature (5 °C), RPB—plastic box, room temperature (21 °C), FPB—plastic box, refrigeration temperature (5 °C); B—egg box type, T—temperature; YW—yolk weight, EYP—yolk proportion in egg weight, YC—colour, YI—yolk index, YpH—yolk pH.

**Table 5:** Fatty acid profile of egg yolk in relation to packaging and storage temperature.

Time (Days)	0	28				Total	SEM	Factor (*p-*Value)		
Treatment		RCB	FCB	RPB	FPB			B	T	B × T
SFA										
C14:0	0.320	0.308	0.348	0.322	0.308	0.321	0.018	*0.337*	*0.064*	*0.661*
C15:0	0.072	0.074	0.070	0.066	0.066	0.069	0.003	*0.806*	*0.185*	*0.425*
C16:0	23.370	19.334	23.512	22.320	22.384	22.184	0.954	*0.582*	*0.013*	*0.504*
C17:0	0.180	0.634	0.162	0.168	0.186	0.266	0.087	*0.439*	*0.716*	*0.057*
C18:0	6.724	5.408	6.524	6.700	6.806	6.432	0.296	*0.858*	*0.760*	*0.639*
C20:0	0.018	0.016	0.014	0.016	0.012	0.015	0.002	*0.630*	*0.923*	*0.772*
MUFA										
C14:1n5	0.068	0.132	0.092	0.074	0.064	0.086	0.014	*0.347*	*0.295*	*0.052*
C16:1n7	2.848	6.846	3.840	3.276	2.650	3.892	0.769	*0.747*	*0.092*	*0.017*
C18:1 n9 c and C18:1 n9 t	38.932	33.114	40.416	42.372	40.190	39.005	1.396	*0.625*	*0.300*	*0.228*
C20:1n9	0.246	0.178	0.224	0.216	0.214	0.216	0.010	*0.289*	*0.450*	*0.257*
C22:1n9	0.004	0.004	0.012	0.004	0.000	0.005	0.002	*0.709*	*0.120*	*0.184*
PUFA										
C18:2 n6 c and C18:2 n6 t	20.608	24.066	17.280	17.166	20.016	19.827	1.005	*0.987*	*0.920*	*0.048*
C18:3 n6 γ	0.078	4.250	0.092	0.078	0.090	0.918	0.832	*0.409*	*0.242*	*0.371*
C18:3 n3 α	0.610 ^ab^	0.360 ^a^	0.804 ^b^	0.728 ^ab^	0.596 ^ab^	0.620	0.049	*0.259*	*0.674*	*0.026*
C20:2 n6	0.232	0.150	0.146	0.144	0.194	0.173	0.012	*0.239*	*0.268*	*0.537*
C20:3 n6	0.102	0.064	0.106	0.094	0.112	0.096	0.008	*0.030*	*0.139*	*0.270*
C20:4 n6	1.412 ^ab^	0.938 ^a^	1.258 ^ab^	1.292 ^ab^	1.494 ^b^	1.279	0.064	*0.040*	*0.025*	*0.457*
C20:3 n3	0.008	0.004	0.012	0.008	0.004	0.007	0.002	*0.756*	*0.605*	*0.263*
C22:2 n6	0.040	0.046	0.054	0.060	0.058	0.052	0.004	*0.414*	*0.114*	*0.280*
C22:6 n3	0.000 ^a^	0.024 ^ab^	0.164 ^ab^	0.182 ^ab^	0.350 ^b^	0.144	0.041	*0.128*	*0.092*	*0.859*

^a,b^–means within row differ significantly at *p* ≤ 0.05; RCB—cardboard box, room temperature (21 °C); FCB—cardboard box (5 °C), refrigeration temperature (5 °C), RPB—plastic box, room temperature (21 °C), FPB—plastic box, refrigeration temperature (5 °C); B—egg box type, T—temperature; C14:0—myristic acid, C15:0— pentadecanoic acid, C16:0—palmitic acid, C17:0—margaric acid, C18:0—stearic acid, C20:0—arachidic acid, C14:1 n5—tetradecenoic acid, C16:1 n7—palmitoleic acid, C18:1 n9 c and C18:1 n9 t— oleic acid, C20:1 n9—eicosenoic acid, C22:1 n9—erucic acid, C18:2 n6 c and C18:2 n6 t—linoleic acids (LA) cis and trans, respectively, C18:3 n6-γ—linolenic acid (GLA), C18:3 n3-α—linolenic acid (ALA), C20:2 n6—eicosadienoic acid, C20:3 n6—dihomo-γ-linolenic acid, C20:4 n6- arachidonic acid (AA), C20:3 n3–eicosatrienoic acid, C22:2 n6—docosadienoic acid, C22:6 n3—docosahexaenoic acid (DHA). SFA—saturated fatty acids, MUFA—monounsaturated fatty acids, PUFA—polyunsaturated fatty acids.

**Table 6:** Fatty acid indexes of egg yolk depending on packaging and storage temperature.

Time (Days)	0	28				Total	SEM	Factor (*p-*Value)		
Treatment		RCB	FCB	RPB	FPB			B	T	B × T
SFA	30.680	31.386	30.630	29.588	29.762	30.409	0.287	*0.567*	*0.055*	*0.417*
MUFA	42.154	42.662	44.584	45.942	43.118	43.692	0.530	*0.699*	*0.507*	*0.086*
PUFA	23.090	22.114	19.916	19.752	22.914	21.557	0.594	*0.658*	*0.742*	*0.096*
n3	0.618 ^ab^	0.514 ^a^	0.980 ^b^	0.918 ^ab^	0.950 ^b^	0.796	0.056	*0.031*	*0.086*	*0.053*
n6	22.472	21.600	18.936	18.834	21.964	20.761	0.590	*0.792*	*0.849*	*0.060*
n9	39.182	39.690	40.652	42.592	40.404	40.504	0.497	*0.643*	*0.298*	*0.221*
PI	29.178	38.570	26.967	26.959	31.726	30.680	2.496	*0.118*	*0.119*	*0.248*
AI	0.374	0.319	0.387	0.360	0.358	0.359	0.016	*0.595*	*0.016*	*0.694*
TI	0.880	0.747	0.864	0.824	0.823	0.827	0.037	*0.311*	*0.040*	*0.321*
DFA	71.968	70.184	71.024	72.394	72.838	71.682	0.344	*0.890*	*0.014*	*0.483*
HSFA	23.626	19.646	23.860	22.642	22.692	22.493	0.968	*0.558*	*0.012*	*0.499*
h/H	2.623	2.495	2.533	2.732	2.761	2.634	0.040	*0.714*	*0.019*	*0.958*

^a,b^—means within row differ significantly at *p* ≤ 0.05; RCB—cardboard box, room temperature (21 °C); FCB—cardboard box (5 °C), refrigeration temperature (5 °C), RPB—plastic box, room temperature (21 °C), FPB—plastic box, refrigeration temperature (5 °C); B—egg box type, T—temperature; SFA—saturated fatty acids, MUFA—monounsaturated fatty acids, PUFA—polyunsaturated fatty acids, PI—peroxidizability index, AI—atherogenicity index, TI—thrombogenic index, DFA—desirable fatty acids, HFSA—hypercholesterolaemic saturated fatty acids, h/H—hypocholesterolaemic*/*hypercholesterolaemic ratio.

## Data Availability

The data presented in this study are available on request from the corresponding author.
